# Unlocking STING as a Therapeutic Antiviral Strategy

**DOI:** 10.3390/ijms24087448

**Published:** 2023-04-18

**Authors:** Annalaura Paulis, Enzo Tramontano

**Affiliations:** Department of Life and Environmental Sciences, Università Degli Studi di Cagliari, 09124 Cagliari, Italy; annalaurapaulis@unica.it

**Keywords:** STING, antivirals, innate immunity

## Abstract

Invading pathogens have developed weapons that subvert physiological conditions to weaken the host and permit the spread of infection. Cells, on their side, have thus developed countermeasures to maintain cellular physiology and counteract pathogenesis. The cyclic GMP-AMP (cGAMP) synthase (cGAS) is a pattern recognition receptor that recognizes viral DNA present in the cytosol, activating the stimulator of interferon genes (STING) protein and leading to the production of type I interferons (IFN-I). Given its role in innate immunity activation, STING is considered an interesting and innovative target for the development of broad-spectrum antivirals. In this review, we discuss the function of STING; its modulation by the cellular stimuli; the molecular mechanisms developed by viruses, through which they escape this defense system; and the therapeutical strategies that have been developed to date to inhibit viral replication restoring STING functionality.

## 1. Introduction

**Figure 1:**
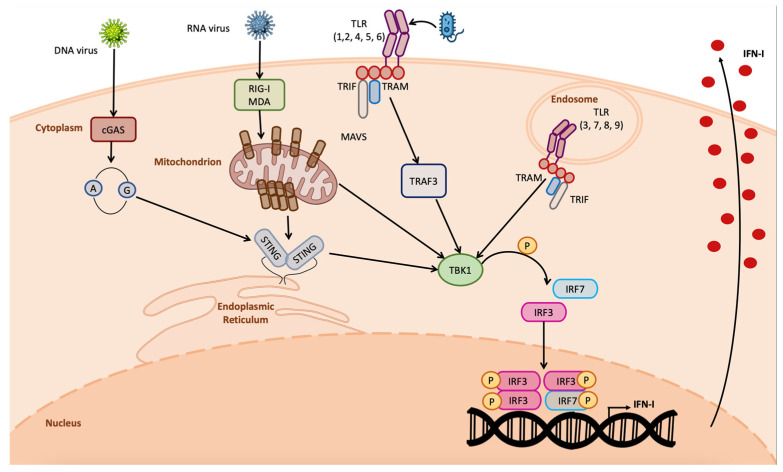
Overview on IFN-I induction by pathogens. DNA viruses are detected by cGAS owing to the DNA release in the cytosol, inducing STING dimerization and IFN-I transcription; RNA viruses are detected by RIG-I and MDA5, inducing MAVS multimerization in the mitochondrial membrane, leading to IFN-I production; TLRs are activated by the detection of bacterial PAMPs, inducing IFNs. All of these pathways require TBK1 kinase activity.

Organisms have learnt how to coexist with invading pathogens by developing barriers that could counteract the invasion and spread of pathogen. The first step for an invading pathogen is to overcome chemical and physical barriers such as the skin, mucous membranes, antimicrobial enzymes, and peptides, which are acidic or basic environments that limit pathogen invasion [1]. However, in some cases, these barriers are not sufficient to counteract the invasion and pathogens can attack the organism, gaining access to the main organism’s delivery system, the blood flux, through which they reach all the body districts. At the same time, they also come into contact with the intrinsic cellular defense system, where they are detected, triggering the activation of the organisms’ countermeasures [1]. The pathogens’ detection occurs through specific proteins called pattern recognition receptors (PRRs), which bind pathogens’ structures such as lipopolysaccharide (LPS), nucleic acids, and pathogens’ proteins; more in general, the so-called pathogen-associated molecular patterns (PAMPs) [2,3]. Different PRRs are involved in the pathogen recognition depending on the PAMP detected, among them are the toll-like receptors (TLRs) and retinoic-acid-inducible gene (RIG)-I-like receptors (RLRs), as well as NOD-like receptors (NLRs) and cyclic GMP-AMP synthase (cGAS) [4,5,6,7] (Figure 1). TLRs are transmembrane receptors located on the cellular surface or on the membrane of intracellular vesicles such as endosomes and lysosomes that detect a wide variety of PAMPs such as lipoproteins, LPS, flagellin, C–phosphate–G (CpG)-DNA, viral ssRNA, and dsRNA. Differently, RLRs, NLRs, and cGAS are all located in the cytoplasm of the cells. RLRs detect long and short dsRNA and 5′triphosphate RNA, inducing pTBK1-dependent IFN-I transcription; NLRs detect peptidoglycan component (iE-DAP) and intracellular muramyl dipeptide (MDP); cGAS recognizes viral dsDNA and tumor-derived DNA, producing cyclic dinucleotides that bind the stimulator of interferon genes (STING), inducing IFN-I transcription [4,5,6,7,8,9] (Figure 1). 

PAMP detection is the starting point of the host organism innate immune response that involves the activation of phosphorylating cascades and leads to the production of cytokines and chemokines. Cytokines are low-molecular-weight (15–20 kDa) proteins or glycoproteins [8]. Among the cytokines, there are specific ones principally involved in the response to viral infections, called interferons (IFNs) for their ability to interfere with viral replication [9]. IFNs can be divided into type I, type II, and the more recently identified type III, each of which triggers a different response interacting with specific receptors [9], [10]. IFN-I comprehends 13 IFN-α isoforms and only one IFN-β, as well as IFN-ε, κ, δ, τ, ω, and ζ. [10]. IFN-II has only one member γ [10], while IFN-III includes λ1, 2, 3, and 4 [11,12]. IFNs, thanks to their potency in inhibiting viral replication, have been studied and used as potential medicines to counteract viral infections such as hepatitis C virus (HCV) and hepatitis B virus (HBV) infection [13]. However, treatment with IFNs is not complication-free: cases of autoimmune disorders such as psoriasis, vitiligo, rheumatoid arthritis, and autoimmune hepatitis have been reported in patients treated with IFNα, indicating that direct exposure to INFs can be comparable to an uncontrolled cytokine expression incompatible with physiologic conditions where the innate immune response is tightly regulated [14].

In the fight for spreading, viruses have evolved strategies to evade the innate immune response at different levels. A number of virus-specific mechanisms through which viruses mask themself to prevent PRR detection have been identified [15]. Viral evolution has indeed occurred by selecting strains possessing proteins capable of interacting with cellular proteins, preventing the transcription factors’ activation or their translocation in the nucleus, impeding the transcription of IFNs or IFN stimulated genes (ISGs) [15,16,17]. Moreover, viral enzymes were shown to be able to degrade cellular proteins by direct cleavage or by regulating the cellular-proteasomal-mediated degradation [18,19,20].

The search for new targets and antiviral agents is continuously growing, especially with broad-spectrum activity. In fact, even though vaccines are the elective way to limit diseases and possibly eradicate pathogens, they are not always effective, particularly in people with compromised immune defenses, and they can also lose activity against rapidly evolving pathogens. Hence, the identification of novel antiviral agents is a priority for health systems [15], and the number of approved antiviral drugs is increasing yearly; up to today, over one hundred antivirals have been approved [21,22]. The most common strategies to develop antiviral drugs are based on the identification of molecules targeting viral proteins and blocking viral replication as direct acting agents. Although successful, this strategy must consider that most viruses are easily capable of selecting drug-resistant strains [22]. Hence, a novel approach for the identification of potential broad-spectrum antivirals is to target cellular proteins inducing an innate immune response, thus avoiding the high mutagenesis rate occurring for viral proteins [23,24,25].

Among the proteins possibly used as drug targets, a recently discovered one is STING—a downstream actor in the detection of non-self cytosolic nucleic acids related to viral infections and tumor conditions [26,27,28,29,30]. STING plays a pivotal role in counteracting viral infections, independently from whether the viral genome is DNA or RNA, mounting a strong innate immune response driven principally by IFN-I [31,32]. On the one side, when cytosolic DNA is detected, cGAS produces cyclic GMP-AMP (2′3′ cGAMP) that directly binds STING, determining TBK1 phosphorylation (pTBK1) and hence transcription of IFN-I [28,32,33]. On the other side, when the RIG-I pathway is activated in response to viral RNA detection, STING interacts with activated mitochondrial antiviral signaling protein (MAVS), determining pTBK1-mediated IFN-I production [32,34]. 

In this review, we will analyze STING interaction with mitochondrial proteins involved in the innate immune response, how mitochondrial DNA release can trigger innate immune response through STING activation, the proteins involved in ubiquitin-mediated STING regulation, and how viruses interact with this pathway to inhibit it. Finally, we will summarize the efforts that have been made in the search for broad-spectrum antivirals targeting STING.

## 2. Functional Role of STING

STING protein is the product of Tmem173 gene; it is a transmembrane protein identified in the first decade of the new century by different research groups simultaneously, starting from a previously uncharacterized molecule supposed to be an inducer of the INF-I response [32,35,36]. STING itself cannot be considered a PRR; in fact, its activation is an event downstream of DNA detection in the cytosol by cGAS that determines the production of 2′3′ cGAMP; hence, cGAS is the actual PRR in the cascade. Cytosolic DNA represents a red flag for the cells. During DNA damage reparation, it commonly happens that short ssDNA fragments are released in the cytosol; DNA repair and replication factors are responsible for pulling back these ssDNA fragments to the nucleus, where they are degraded by endonucleases [37,38]. When this mechanism is altered, the ssDNA accumulation in the cytoplasm induces cGAS activation, leading to the production of cyclic dinucleotides (CDNs) [38]. Of note, only dsDNA with a minimum length of 36 nucleotides can effectively induce cGAS, as short dsDNA fragments bind to cGAS in a manner not stable enough to induce the formation of CDNs [37,38].

**Figure 2:**
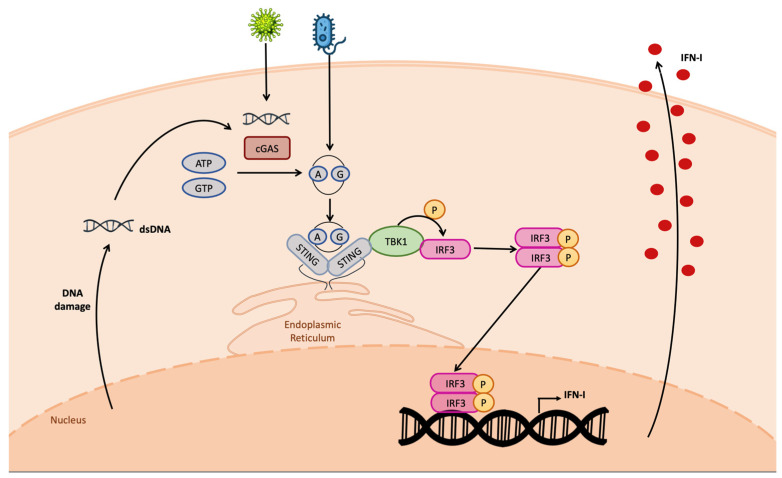
cGAS–STING pathway. The cGAS protein detects cytosolic DNA, leading to the production of CDNs. Both cellular- and bacterial-derived CDNs bind STING, leading to the activation of TBK1 kinase activity on IRF3. Phospho-IRF3 dimer enters the nucleus, binding the IFN-I promoter, inducing IFN-I transcription. IFN-I is produced and secreted outside the cell.

The cGAS-produced CDN in turn directly binds STING, leading to its dimerization and phosphorylation (Figure 2). Once activated, STING determines TBK-1-mediated IRF3 phosphorylation; pIRF3 dimerizes; and the dimer translocates in the nucleus, establishing the antiviral state [39]. 

Comparative analysis between wild-type cells expressing STING and STING^-/-^ cells demonstrated that knocking out of STING reduced IFN-β as well as pro-inflammatory cytokines’ production, resulting in higher susceptibility to viral and bacterial infections [40,41,42]. The same pathway was also found to be activated during infections with *L. Monocytogenes*, an intracellular pathogen that expresses di-adenylate cyclase (DAC), which produces the CDN 3′3′ cGAMP, detected by STING, inducing IFN-β production. Interestingly, this enzyme was identified through bioinformatic analysis in bacteria and archaea including *Staphylococci*, *Streptococci*, *Mycobacteria*, *Chlamydia*, and *Mycoplasma* spp., possibly suggesting that all of these pathogens could induce a STING-mediated immune response [43].

The cGAS-mediated pathway is not the only possible STING activation pathway. In fact, several studies reported that, when viral RNA in the cytosol is detected by RIG-I or MDA5, depending on whether the RNA is short or long, respectively [4], STING may contribute to the RIG-I-mediated innate immune response through direct interaction with RIG-I, but not MDA5, as STING is not able to interact with MDA5 [44]. Of note, as confirmation of the importance of STING in infection with RNA viruses, RNA viruses have evolved strategies to target the STING protein in order to overcome the innate immune response [32,45,46,47].

In addition, it was also demonstrated that long-term exposure to the genotoxin etoposide induces the activation of DNA damage inside the nucleus, leading to a non-canonical STING activation with neither cGAS activation nor cGAMP production. In this case, the proteins ATM (ataxia telangiectasia mutated) and PARP1 (poli-ADP-ribose polymerase 1) are responsible for p53 phosphorylation, which leads to the interaction with IFN-inducible factor 16 (IFI16) in the nucleus, leading to the interaction with the cytosolic E3 ubiquitin ligase tumor-necrosis-factor-receptor-associated factor 6 (TRAF6). This complex, assembled straddling the nuclear membrane, interacts with STING located on the ER surface, leading to its TRAF6-mediated K-63-linked poli-ubiquitination [48,49]. The non-canonically activated STING preferably activates NF-kB rather than IRF3, inducing a different gene profile transcription with respect to the canonical STING activation [48].

## 3. Structure of STING and Interaction with Ligands

**Figure 3:**
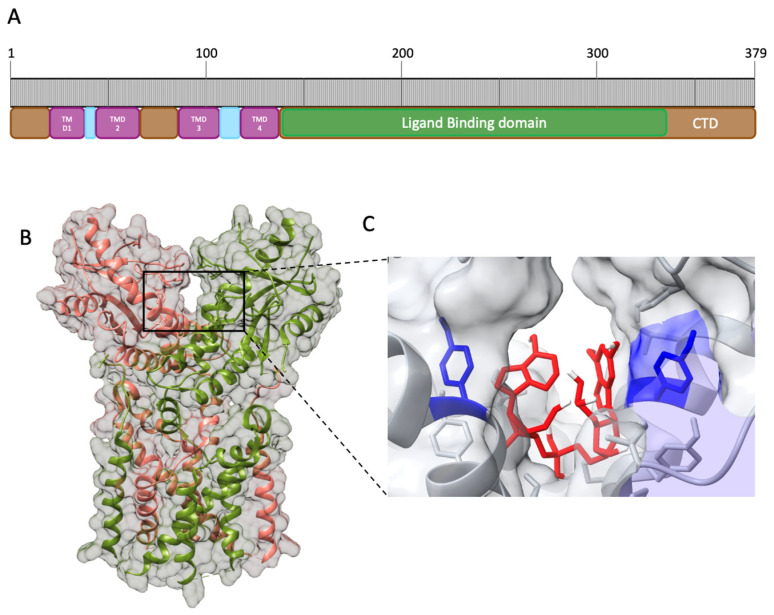
Human STING (hSTING) secondary structure and CDN binding site. (**A**). Schematic representation of the apo hSTING secondary structure. (**B**). Open conformation of hSTING homodimer (PDB: 8GT6). (**C**). Prediction of 2′3′ cGAMP (red) interaction with hSTING Tyr 167 residues (blue) obtained through molecular docking of hSTING (8gt6) with 2′3′ cGAMP extracted from the deposited chicken STING structure (PDB: 5GRM), obtained with AutoDock 1 [52] and Discovery Studio Visualizer [53].

STING is composed of 379 amino acids (aa) that can be divided into nine domains (Figure 3A). Starting from the ammino-terminal portion, STING presents four transmembrane domains (TMD) responsible for the localization in the organelles’ membrane, endoplasmic reticulum, and mitochondrion. The main domain, comprehending aa 139–379, is the cytosolic domain (CTD), located in the carboxyl-terminal region in which reside the ligand-binding domain (LBD) and the highly conserved dimerization motif GXXXS required for STING dimerization; the remaining domains are considered linker domains [50]. STING exerts its function through homodimerization by adopting the α+ β fold, involving five-stranded sheets in the center and four helices in the periphery [50] (Figure 3B). The dimerization motif localized in the transmembrane region identified as LBDα1 is essential for STING activity, as demonstrated through mutagenesis studies [51]. The resulting homodimer is the functional element able to bind the signaling nucleotide 2′3′ cGAMP. Its binding site is located in the interface between the two chains, where the Tyr167 in the LBDα1 is responsible for the binding with 2′3′ cGAMP (Figure 3C). 

The binding requires charge–charge interaction between the phosphates in the 2′3′ cGAMP and the protein residues and determines the conformational change from an open to a closed active form [51,54,55,56,57]. Of note, bacterial CDN, 3′3′ cGAMP, is also detected by STING; however, it can only form a weak interaction with STING in the binding site, suggesting lower effectiveness in the activation of STING with respect to the interaction with the 2′3′ cGAMP, indicating that STING is not the major detector of bacterial infection, but is still involved in building the innate immune response against bacteria too [43,58,59]. STING interacts with TBK1 through its cytosolic domain, more specifically, with the LBD, in a constitutive manner. The binding with 2′3 cGAMP induces STING multimerization, leading to trans-autophosphorylation of TBK1 [60]. pTBK1 attracts interferon regulatory factor 3 (IRF3), forming the STING–TBK1–IRF3 complex. Although the complex formation dynamics are still unclear, the interaction leads to IRF3 dimerization and phosphorylation mediated by pTBK1 [50]. Then, the active IRF3 dimer translocates in the nucleus, where it binds the IFN-I promoter, leading to IFN-I production [36,61,62].

## 4. STING Connection with Mitochondria

Mitochondria are cellular organelles with a significant role in cellular metabolism, as they are the center of ATP production and serve as metabolic hubs. They are factories for the biosynthesis of macromolecules as lipids, proteins, and nucleotides [63], and they also play a pivotal role in redox homeostasis responding to cell’s stressors, both cellular and environmental, as well as in programmed cell death, responding to pathogenic conditions leading to instauration of a pro-apoptotic state or triggering the activation of the innate immune response [63,64,65].

**Figure 4:**
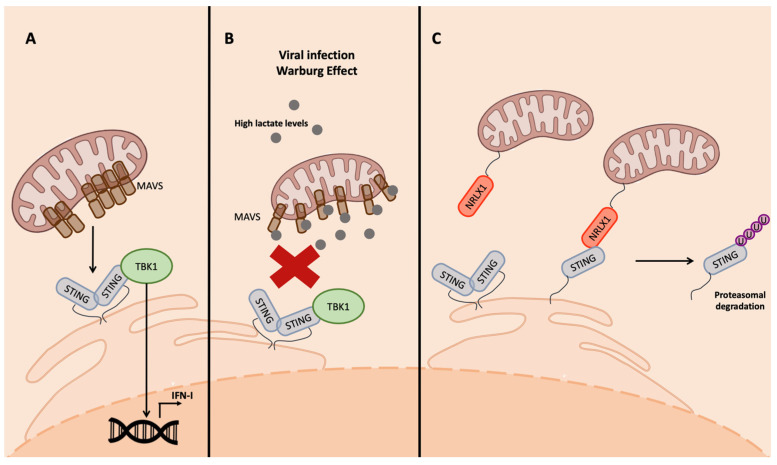
Inhibition of STING interaction with MAVS. (**A**) MAVS interaction with STING is required for the activation of RIG-I-mediated IFN-I transcription. In physiologic conditions, MAVS aggregate in the mitochondrial surface and bind STING, leading to its aggregation and activation of IFN-I transcription. (**B**) Viral infection, as well as tumorigenesis, can lead to the production of lactate as a bioproduct of aerobic glycolysis; lactate binds MAVS that cannot aggregate and, as a consequence, the MAVS–STING interaction is impeded. (**C**) NLRX1 in viral-infected cells inhibits the MAVS–STING interaction through direct binding with SITNG, leading to its proteasomal degradation.

One of the most important mitochondrial proteins involved in the innate immune response is MAVS, a transmembrane protein located in the outer membrane (OM) of the mitochondria. MAVS is an effector of the RIG-I pathway, involved when RNA viruses are detected. MAVS aggregates and interacts with STING to induce TBK1 phosphorylation and then IFN-I transcription [63] (Figure 4A). MAVS is also modulated by bioproducts of the metabolism. In fact, it is associated in the OM with the glycolytic enzyme hexokinase 2 (HK2), which prevents MAVS multimerization in normal conditions when RLRs are not activated [63,66].

Viruses have developed strategies to manipulate mitochondria metabolism to inhibit IFN production. An interesting example is the small molecule lactate [63], a bioproduct of the lactate dehydrogenase complex (LDH) that generates it, starting from acetyl-CoA in absence of oxygen. This production can actually occur even in presence of oxygen because of the aerobic glycolysis, a condition called the Warburg effect, which is frequently promoted during viral infections and in cancer cells [62,67,68,69]. Lactate then binds MAVS, preventing its aggregation and the interaction with the downstream effectors of the cascade involved in IFN-I production, TBK1 and STING [63] (Figure 4B).

Another important mitochondrial protein that interacts with the STING signaling pathway is the nucleotide binding oligomerization domain (NOD)-like receptor 1 (NLRX1). It belongs to the NLR family; is ubiquitously expressed; possesses the NACHT domain, responsible for hetero-dimerization, and the leucine-rich repeats domain (LRR) in the C-terminus; and is the only NLR localized on the OM owing to the presence of a mitochondrial targeting sequence in the *N*-terminus [4,70]. This localization was reported to be important for the interaction with proteins involved in innate immunity, such as the mitochondrial MAVS and STING, as well as cytoplasmic proteins such as TRAF6 and IKK complex [4,71].

The interaction of NLRX1 with the effectors of the innate immune response occurring through the NACHT domain promotes their ubiquitination and, consequently, proteasomal degradation, resulting in the inhibition of NF-kB and IRF signaling following viral infection [71] (Figure 4C). 

In fact, in cells infected with Sendai virus, influenza A virus, and HCV, the RIG-I/MAVS signaling was negatively affected by NLRX1 [70,71]. This inhibition is related to glucose levels; high glucose levels activate NLRX1, which binds poly(rC) binding protein 2 (PCBP2), which drives MAVS, unable to aggregate owing to the presence of a lactate covalent bond, as previously described, to ubiquitination and degradation [70,71]. A very similar mechanism was reported for STING in cells infected with human immunodeficiency virus type-1 (HIV-1), in which reverse-transcribed viral DNA induces IFN-I transcription through cGAS-STING; in human papilloma virus 16 (HPV16)-infected cells, the viral protein E7 promotes NLRX1-mediated STING degradation [72,73].

## 5. Triggering of STING through mtDNA Release

**Figure 5:**
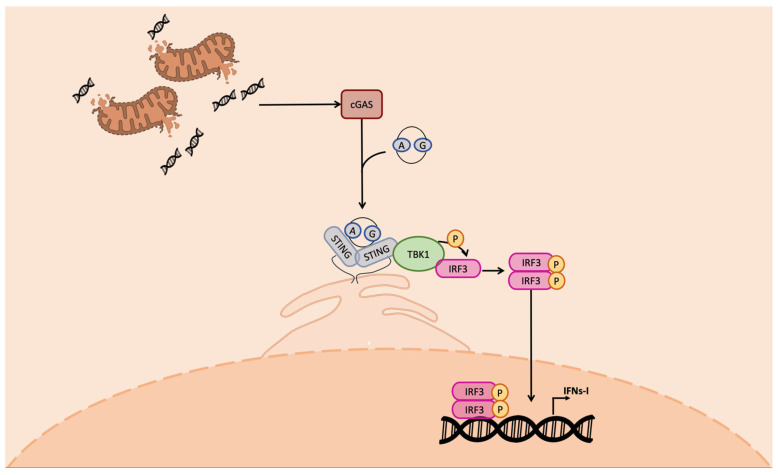
mtDNA stress activates the cGAS-STING pathway. During viral infection, mitochondria undergo the induction of a stressed state, during which the mtDNA nucleoids lose their compacted state and are released into the cytoplasm, where mtDNA is detected by cGAS, activating the cGAS-STING pathway.

The mitochondrial genetic system, constituted of a double-stranded circular DNA, was identified when studying the various encoded proteins mostly involved in the oxidative phosphorylation [74]. It is organized in highly controlled and regulated protein-rich structures and it is present in at least one copy per mitochondrion, thus resulting in hundreds of copies per cell. The loss of this highly controlled structure is called “mtDNA stress” and leads to the release of mtDNA in the cytosol [75]. The mtDNA in the cytosol is detected as foreign DNA, triggering cGAS-STING-IRF3-dependent IFN-I transcription and, consequently, ISGs’ transcription [75] (Figure 5). 

Viruses interact with mitochondria and mtDNA in different ways. Herpes viruses have been reported to induce mtDNA stress and, consequently, its release in the cytosol, activating the STING pathway. In particular, the alphaherpesvirus protein UL12.5 localizes in the mitochondria, where it contributes to the mtDNA depletion often coupled with its release. Indeed, the absence of this viral protein was found to mount a less potent TBK1 STING-dependent phosphorylation with respect to the wild type, confirming an mtDNA-dependent innate immune response [75]. Dengue virus (DENV) infection induces RIG-I- and MDA5-mediated IFN response. It also determines cellular damage causing mtDNA release and inducing IFN-I transcription in a cGAS-STING-dependent way. As a countermeasure, DENV produces the NS2B protease, which cleaves cGAS, neutralizing its activity [76].

Mitochondria also play a key role in nucleotide biosynthesis as a central hub for the dNTP salvage synthesis pathway [63]. Briefly, the multifunctional protein CAD, comprehending the carbamoyl-phosphate synthetase (CPSase), the aspartate transcarbamylase (ATCase), and the dihydroorotase (DHOase); the dihydroorotate dehydrogenase (DHODH) located in the inner mitochondrial membrane; and uridine monophosphate synthetase (UMPS) are responsible for de-novo pyrimidine biosynthesis, starting from glutamine, aspartate, and bicarbonate; meanwhile, the salvage pathway processes uridine and cytidine in order to produce intermediates embedded in the de novo nucleotide synthesis cascade [63,77]. Some enzymes involved in pyrimidine biosynthesis such as DHODH and CAD were found to be targeted by antiviral compounds, leading to pyrimidine deprivation, impairing viral genome production and, as a consequence, viral replication [77,78,79,80,81,82,83,84]. Moreover, interestingly, pyrimidine deprivation is related to mtDNA release and, as a consequence, to the activation of a cGAS-STING-mediated IFN response [78,85]. However, it is still unclear whether this mechanism takes place in infected cells as an antiviral defense, inducing spontaneous mtDNA release to trigger innate immunity.

## 6. Role of Ubiquitin in the STING-Mediated Innate Immune Response

The ubiquitin proteasome system comprehends ubiquitin (Ub), a highly conserved small molecule of 76 amino acids and 3 enzymes: the E1 Ub-activating enzyme, the E2 Ub-conjugating enzyme, and the E3 ligase [86,87]. The best-known function of Ub conjugation is to target proteins to modulate their half-life, driving target proteins to proteasomal degradation; however, a second role is the regulation of protein activity [86,87,88]. The Ub chain is bound to the target protein starting from its activation: E1 adenylates the Ub C-terminal group; then E2 transfers the activated Ub to the active site; and finally E3 catalyzes the formation of a covalent bond between a lysine, belonging to the target protein, and the Ub C-terminal [86].

Ubiquitination can occur as mono- or polyubiquitination in selected Ub lysine residues: K6, K11, K27, K29, K33, K48, and K63 [89]. Only two of them, K29 and K48, are related to proteasomal degradation; all of the others modulate protein activity during inflammation, innate immune response, endocytic trafficking, transcriptional regulation, and DNA repair [90,91,92]. These Ub chains can be removed from target cells through the activity of enzymes called deubiquitinases (DUB or USP) [93]. Ubiquitination and deubiquitination are tightly regulated because the absence of regulation in processes as important as the innate immune response could lead to pathogenic conditions like immune diseases [94]. 

STING activity is controlled and regulated through Ub conjugation and deconjugation; in fact, binding to CDNs is sufficient to induce the conformational changes required for STING multimerization and IFN-I induction; however, ubiquitination can enhance its activity or regulate the protein’s half-life [95].

**Figure 6:**
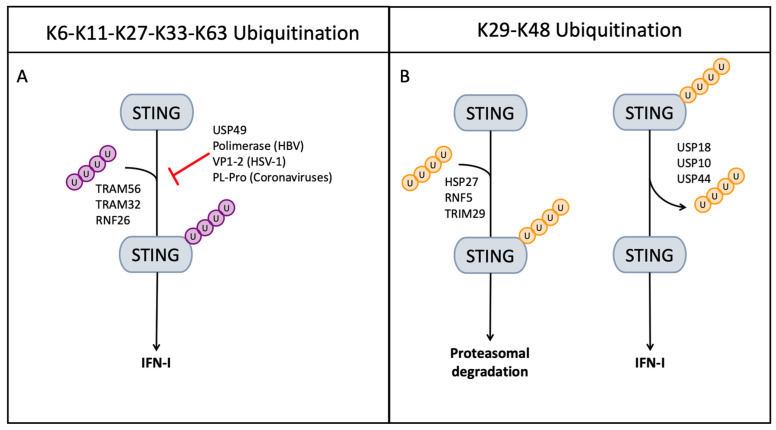
STING ubiquitination. (**A**) K6–K11–K27–K33–K63 ubiquitination is required to induce STING activation and IFN-I transcription; viral proteins as well as cellular proteins inhibit the IFN response by deubiquitinating or inducing deubiquitination. (**B**) K29–K48 ubiquitination is the signal for proteasomal degradation; in viral-infected cells, this ubiquitination is promoted and the cellular proteins HSP27, RNF5, and TRIM29 ubiquitinate STING in order to induce proteasomal degradation; K29–K48 deubiquitination is promoted in order to establish the antiviral state with the activity of USPs.

Enhancing ubiquitination requires the activity of two of the main E3 ligases, tripartite motif (TRIM) protein 56 and 32, which have been found to link K-63 ubiquitin to STING. TRIM56 ubiquitinates K150 residue, while TRIM32 ubiquitinates multiple lysine residues (20/150/224/236) (Figure 6A) [96]. The E3 ligase ring finger protein 26 (RNF26) mediates K11 ubiquitination, protecting STING from K48 Ub conjugation and preventing its degradation [97] (Figure 6A). 

The proteasomal degradation, on the other hand, is induced through K-29 and K-48 ubiquitination; the RNF5, TRIM29, and the heat shock protein 27 (HSP27) mediate K-48/K-29 UB conjugation, leading to STING degradation [98,99,100] (Figure 6B). Cellular USPs modulate the STING ubiquitination by removing UB chains: USP18, USP20, and USP44 target STING, promoting K-48 deubiquitination and preventing STING degradation; meanwhile, USP49 was found to play a pivotal role in negatively regulating cellular antiviral responses via deconjugating K63-linked ubiquitination of STING [96,101,102,103] (Figure 6B).

Small Ub-like modifier conjugation (SUMOylation) is comparable to ubiquitination as it modulates protein activity, function, stability, subcellular localization, and the interaction with other proteins [104]. Four different SUMOs are known: SUMO1-2-3-4; SUMO 2 and 3 are isoforms, SUMO1 shares 50% homology with SUMO2/3, while SUMO4 is the least studied. The conjugation mechanism for Ub requires the maturation of SUMO, E1-mediated SUMO activation, E2-mediated conjugation, and E3-mediated ligation [104]. The TRIM38 is a E3 ligase found to ligate SUMO to both cGAS and STING in uninfected cells and in the early phases of infection, regulating their activity. However, both cGAS and STING were de-SUMOylated by sentrin specific peptidase 2 (Senp2), leading to their degradation [105].

Viruses, on their hand, have evolved proteases and mechanisms to mediate either Ub conjugation or deubiquitination in order to modulate intrinsic cell processes to their advantage. HBV possesses two proteins that effectively block cGAS and STING, the viral polymerase deubiqutininates K-63 STING and downregulates dsDNA sensing, while the HBx protein downregulates the accumulation of cGAS expression by promoting its ubiquitination and autophagy [106,107]. In herpes simplex virus 1 (HSV-1)-infected cells, cellular USP49 as well as HSV-1 protein 1-2 (VP1-2) have deubiquitinatig activity against STING targeting K-63 linked Ub inhibiting its activity [108]. In cells infected with HSV-1, the cellular protein RNF5 plays a major role in inhibiting the STING-mediated innate immune response by K-48 ubiquitination [96]. In cells infected with pseudorabies virus (PRV), a herpesvirus causing diseases in domestic and wild animals, HSP27 promotes PRV proliferation by targeting the cGAS-STING pathway, inhibiting IFN-I transcription [100]. In Epstein–Barr-virus-infected cells, TRIM29 ubiquitinates STING, inducing its proteasomal degradation to inhibit the innate immune response [99]. All coronaviruses, (+) ssRNA viruses, encode for at least one papain-like protease (PLP) that acts as DUB removing Ub chains from target proteins; moreover, the intrinsic characteristics of these proteases make them able to cleave interferon-stimulated gene 15 (ISG15), an ISG with similar activity to Ub [34,109,110].

## 7. Direct Viral STING Inhibition

STING is also directly targeted by viral proteins, determining its degradation or inactivation. HSV-1 encodes three different proteins that inhibit STING signaling: ICP27 interacts with the activated STING–pTBK1 complex, preventing phosphorylation and activation of the transcription factor IRF3 [111]; UL46, a tegument protein, binds both STING and TBK1 with different domains, preventing STING and TBK1 interaction [112]; and γ134.5 prevents STING phosphorylation by direct interaction [113]. STING plays a major role in Kaposi sarcoma virus (KSHV) detection during primary infection and in the reactivation from latency with the viral protein vIRF3 shown to interact with STING, preventing its binding to TBK1 [114]. The HCMV tegument protein, UL82, was found to inhibit STING trafficking towards the Golgi, blocking IFN-I activation [115]. More recently, it was described that the viral protein UL42 inhibits the cGAS-STING pathway, directly interacting with cGAS and STING [116]. Moreover, HCMV possess the IE86 protein that promotes RNF5 activity, determining STING proteasomal degradation [117]. In HCV infection, the viral NS4B protein disrupts STING interaction with TBK1 and MAVS to prevent the activation of the innate immune response [45,118,118]. The same mechanism was reported for DENV NS4B protein. Moreover, the DENV protease NS2B3 has been reported to cleave both cGAS and STING to block IFN-I production [46,47,76].

## 8. STING Agonists as Broad-Spectrum Antivirals

The involvement of STING in activating INF production as well as all of the efforts that viruses put into shutting down the activity of STING clearly indicate that STING is a potential target for small molecules acting as STING agonists that may be broad-spectrum antiviral agents. Indeed, computational studies have confirmed that STING is a druggable target, showing the possibility of accommodating small molecules acting as STING agonists. A few studies have already been published on this topic.

**Figure 7:**
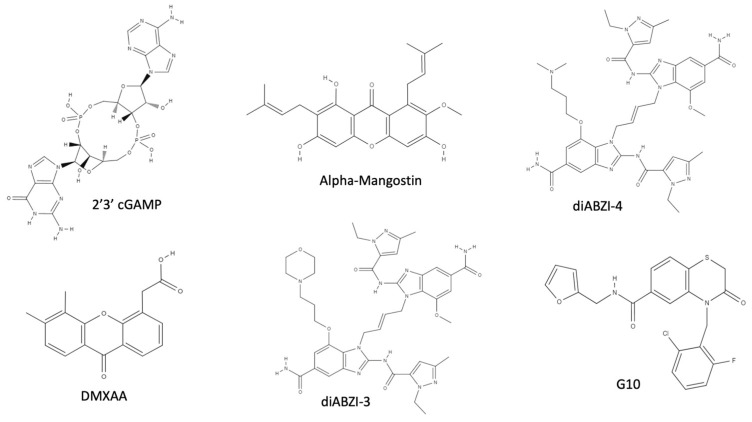
Chemical structure of STING agonists. 2′3′ cGAMP; DMXAA; alpha-Mangostin; diABZI-3; diABZI-4; and 4-(2-chloro-6-fluorobenzyl)-N-(furan-2-ylmethyl)-3-oxo-3,4-dihydro-2H-benzo[b][1,4]thiazine-6-carboxamide, also known as G10.

The second messenger 2′3′ cGAMP (Figure 7) has been studied as a STING natural ligand in the treatment of viral infection because of its potential antiviral activity. In both in vitro cell-based infection and in mice infected with Herpes simplex virus 2 (HSV-2), local and systemic delivery of 2′3′ cGAMP induced strong IFN-I and ISG production. Of note, 2′3′ cGAMP showed stronger antiviral effect with respect to TLR agonists [119,120,121]. However, despite their strong IFN induction, CDNs are not suitable for drug therapy because of their poor membrane permeability and metabolic instability [29,122].

DMXAA (Figure 7) is a well-known antitumor agent used to induce the disruption of the tumor vasculature and the release of chemokines by the activation of tumor-associated macrophages [123]. Although, once moved to human clinical trials, it failed to show any effect against human tumors. The compound was also demonstrated to be a potent antiviral in murine and mouse models, but showed no effect in human cells; further studies demonstrated that DMXAA selectively binds murine and mouse STING, but not human STING, explaining the inefficacy of the compound in human models [124]. This selectivity resides in the difference among human and mouse STING CTDs; in fact, although mSTING and hSTING binding pockets are composed of identical amino acids, they only share a 76% homology sequence in the CTD, which confers different conformations to the lid domains [57]. Indeed, Gao et al. demonstrated through mutagenesis studies that hSTING Ser162, Glu232, and Gly266 residues are responsible for DMXAA mSTING selectivity over hSTING [125].

Alpha-Mangostin (Figure 7) is a xantone with antimicrobial properties that was reported to induce IFN-I production in a STING-dependent manner, binding to the STING CTD. Alpha-Mangostin has been shown to have antiviral properties and activity against DENV and HBV replication in cell-based assays, while in vivo studies have not yet been performed [126,127,128,129,130].

The dimeric amidobenzimidazoles (diABZIs) are non-CDN STING agonists identified through in silico studies, which showed the ability to induce IFN-β, α-chemokine CXCL1 and IL-6 transcription, and anti-tumor activity [131]. Two of them have been tested for their ability to inhibit viral replication: di-ABZI-3 (Figure 7) was tested in primary human bronchial epithelial cells infected with parainfluenza virus 3 and rhinovirus, subverting viral infection in a STING-dependent manner [132]. More recently, it was active in inhibiting the replication of HCoV-OC43 and SARS-CoV-2 [133,134,135]. di-ABZI-4, which differs from di-ABZI-3 because of its more favorable solubility profile, was also tested in vitro and in an animal model for its ability to inhibit SARS-CoV-2 replication [136].

The result of a high-throughput in vitro screen identified 4-(2-chloro-6-fluorobenzyl)-N-(furan-2-ylmethyl)-3-oxo-3,4-dihydro-2H-benzo[b][1,4]thiazine-6-carboxamide, also called G10 (Figure 7), which was found to active in inhibiting the alphaviruses Chikungunya virus (CHIKV), Venezuelan equine encephalitis virus (VEEV), and Sindibis virus (SINV) in human cells [137].

## 9. Conclusions

During the last few decades, a new protein named STING, involved in the innate immune response, was discovered and characterized [32,35,44]. STING plays a central role in the innate immune response, mediating the IFN production in response to cytosolic DNA (viral, tumor-derived, and mitochondrial DNA) and acting as a linker among the different responses to RNA viruses and bacteria [28,34,48,138]. 

IFNs represent a very strong wake-up call for the organism, meaning that the danger occurring in the cells evokes the activation of defense mechanisms. It is well known that dysregulation in the production of IFNs and their downstream activity can lead to the development of autoimmune diseases. Hence, pathways leading to the production of IFNs must be tightly regulated [14]. STING is not exempted in this fine-tuned regulation. In fact, its activity is modulated by cellular proteins through Ub conjugation and deconjugation in order to control its activation and proteasomal degradation, as well as trafficking between ER and mitochondria, where it interacts with several mitochondrial proteins [88,90,96,98,102]. As the mitochondria is an important hub for cellular metabolism, the interaction with mitochondrial proteins, influenced by metabolism alterations, suggests that STING regulation is also related to metabolism perturbation [66,69,71,72].

Viruses are well known for their ability to mutate to adapt to and overcome cellular defense weapons. Viral proteins target cellular proteins to cleave them entirely or partially by removing post-translational modification required to modulate the protein’s activity, such as, for example, through deubiquitination [18,19,20,100,102,106,139,140,141,142,143]. STING is targeted by both DNA and RNA viruses as it is recognized as a driver for the instauration of the antiviral response; moreover, modulation of cell metabolism alters the ability of STING to interact with upstream and downstream effectors involved in IFNs’ production pathways.

The request for novel antivirals is continuously increasing, especially owing to the rapid onset of novel viral strains leading to the identification of targets among the cellular proteins involved in cellular defense for the development of broad-spectrum antivirals [21,144]. STING’s central role in innate immunity and the viral-mediated inhibition attracted attention to this protein as a potential cellular target for the development of broad-spectrum antivirals. A few molecules identified by computational approaches have been studied in vitro, showing the ability to induce a strong IFN response and, in some cases, to inhibit viral replication in a STING-dependent manner [29,31,31,122,124,127,128,129,131,145,146,147]. Notwithstanding the promising results obtained in vitro, none of the identified compounds active on human STING have been tested in animal models.

Targeting STING remains a suitable strategy to inhibit viral replication by small molecules; further studies are required to identify novel and more effective STING agonists as broad-spectrum antiviral agents.

## Data Availability

Not applicable.
